# Signifying the Effect of Relational and Experiential Cognitive Styles on Entrepreneurial Behavior: A Mediated Moderated Model

**DOI:** 10.3389/fpsyg.2021.762403

**Published:** 2021-10-15

**Authors:** Muhammad Aamir, Muddassar Sarfraz, Kausar Fiaz Khawaja, Habiba Usman, Zhihua Hu

**Affiliations:** ^1^Department of Computer Science, Huanggang Normal University, Huanggang, China; ^2^Binjiang College, Nanjing University of Information Science and Technology, Wuxi, China; ^3^Research Center for Engineering and Management, Politehnica University of Timisoara, Timisoara, Romania; ^4^Faculty of Management Sciences, International Islamic University, Islamabad, Pakistan

**Keywords:** informal learning, entrepreneurial intention (EI), entrepreneurial behavior, entrepreneurial psychology, students

## Abstract

This study investigates the influence of rational and experiential cognitive styles on entrepreneurial behavior. Specifically, the moderating role of entrepreneurial intention and informal learning has been contemplated. Data has been accumulated from 320 undergraduate students of universities situated in Pakistan. Statistical Package for the Social Sciences (SPSS) and AMOS have been executed to examine the data and conduct statistical techniques. After confirming the validity and reliability of data and scale, results have signified that both cognitive styles significantly positively impact entrepreneurial behavior. Moreover, informal learning moderates the relationship between entrepreneurial intention and cognitive styles (rational and experiential). Meanwhile, entrepreneurial intention mediates the relationship between cognitive styles (rational and experiential) and entrepreneurial behavior emphatically.

## Introduction

The COVID-19 pandemic was unpredictable and posed a significant challenge for education management and subjects of a practical/experiential nature (Brammer and Clark, 2020; Ratten and Jones, 2021). According to Clark et al. (2020), countries have updated their regulatory policies to address COVD-19 crises, such as societal disruption and career shock. Although restrictions have focused on social gatherings and maintaining social distancing, this has also affected the job market. Hence, along with the typical basic skills and knowledge, employers now expect young graduates to have the entrepreneurial spirit required to cope with this changing environment.

All university undergraduates and graduates face two career paths when starting their professional life: working for themselves or others. Either way, they face difficulties in deciding how and when to join an organization or start a firm. Large firms with a long-term hiring vision are becoming out of reach for many graduates, and advanced education is no longer a guarantee of a job in the higher levels of a firm. Employees with more than one occupation simultaneously are rising and often preferred. That is likely why the enrollment of students in entrepreneurship programs has increased; it may help to build their entrepreneurial perspective, understanding, and behavior (Molaei et al., 2014).

In an advanced workplace, employees develop novel thoughts, innovations, administrations, and guidelines faster than before. Today, responsive and intelligent firms prefer individuals who do not simply bring abilities and knowledge to the working environment but also can learn, share, and create new knowledge at work. Studies have reported the significance of having informal learning in the workplace (Berg and Chyung, 2008; Jeong et al., 2018). However, few findings have focused on how informal learning moderates between cognitive styles and intention among students inside most widely recognized universities of Pakistan.

Small midsize enterprises (SMEs) are different from large organizations in terms of their employees, organization structures, assets and abilities, administrative, leadership styles, and greater exposure to the external environment (Man et al., 2002). Hence, they need to concentrate on their assets and styles of individuals to use informal learning and develop entrepreneurial behavior. SMEs arguably have more to gain by using an connections of an individual and participation in informal learning than more prominent organizations (Xerri and Brunetto, 2011). For potential entrepreneurs like university graduates, entrepreneurship starts from an idea followed by practices, including the adequacy of thoughts and later gathering of information to evaluate fresh ideas (Hayton and Cholakova, 2012). Hence, entrepreneurial intention of university students is based on entrepreneurial thoughts and ideas and informal learning that later formulates entrepreneurial behavior. Therefore, a lack of entrepreneurial intention may arise from a lack of related thoughts and learning. Based on these arguments, the objective of this study is to investigate the mediating role of entrepreneurial intention between cognitive styles and entrepreneurial behavior, and the moderating role of informal learning between cognitive styles and entrepreneurial intention. Section 2 presents a literature review and hypotheses development; Section 3 presents research methodology; Section 4 presents study results; Section 5 presents discussion; and Section 6 presents conclusion.

## Literature Review and Hypotheses? Development

### Theory of Planned Behavior

The theory of planned behavior (TPB) proposes three autonomous determinants of intention. The attitude toward the behavior indicates significant, undesirable assessment or evaluation of the behavior of a person. The second indicator is a social factor labeled the subjective norm; it shows the perceived societal stress to manage or not to manage the behavior. Perceived behavioral control is the third indicator of intention, and it indicates the difficulty of managing the behavior in terms of perceived comfort. It is expected that, on the way, previous knowledge will be redirected, as expected hindrances and restrictions. Overall, the more positive the approach and emotional standard concerning behavior, the more robust perceived behavioral control is. The intention of a person should be grounded to manage the attitude and measure the intention to shift across behaviors and circumstances. Therefore, it is considered that attitudes fundamentally affect intentions. On the other hand, attitudes and perceived behavioral control represent intentions, and so the three indicators have autonomous influences. The TPB originates from the theory of reasoned action, or the theory of expansion (Fishbein et al., 1980).

As in the initial theory of reasoned action, a significant indicator in the TPB structure is a intention of a person to manage a certain behavior. Intentions are accepted to be a means by which to capture the inspirational components that impact behavior. They indicate that it is difficult for an individual to attempt the specific task others want to be completed while managing a particular behavior. It is considered that individual performance will be improved by having positive behavior. It must be flawless; if the behavior is under voluntary control, the behavioral intention can find emotions in behavior, i.e., if a person decides about the behavior, either they manage it or not. Ajzen (1991) finds that some behaviors may meet this necessity very well; the performance depends on many non-inspiring elements like the availability of needed assets and freedom. All these elements enhance the real power of individuals to accommodate the behavior. If an individual has the necessary assets and more chances through an individual plan to manage the behavior, the individual will do so.

The TPB structure is generally acknowledged as a method for considering entrepreneurial intention, which develops behavior (Schlaegel and Koenig, 2014). The theory focuses on cognitive styles of an individual, which indicates the cognitive capability of an individual who wants to become an entrepreneur. Based on the theory of planned behavior (Schlaegel and Koenig, 2014), it is apparent that individuals have different cognitive styles. Although an individual should select one cognitive style by choice, in general, each style has a different impact on human judgments. It all depends on the difficulty of the work, environment, and individual contracts.

### Cognitive Style and Entrepreneurial Behavior

Rational cognitive style is defined as how people observe from their surroundings and how they coordinate and practice their knowledge from their surroundings to control their activities. Bouckenooghe et al. (2005) pose some key questions on this topic: How do we know cognitive style of an entrepreneur? Is the manner in which they observe, utilize, and arrange their knowledge changed from how normal people do? The findings of their study confirm that entrepreneurs vary in their cognitive styles and develop behaviors accordingly. Successful entrepreneurs like to be innovative, be innovators, and face challenges, as do inventors. People who have experiential cognitive styles search for evidence and information and the need to recognize the truth. They have a habit of storing several pieces of evidence and information. They are more focused on their work and are precise, and they like to grapple with complex issues to find a perfect solution. They are also described as complete and theoretical thinkers. In general, they are innovative and enjoy experimentation, tend to see new beginnings and difficulties, and do not care for rules and methods, and enjoy vulnerability and opportunity. Notably, they are aspiring and achievement-focused. Effective entrepreneurs show more creativity than others and can deliver solutions that counter recognize knowledge. Bridge and O'Neill (2012) report that people who have experiential cognitive styles work to acknowledge professional opportunities. Hodgkinson and Sadler-Smith (2003) state that all cognitive styles are needed to deal with information and limit the risks of cognitive differences that scholars specify as a part of behavioral decisions (Sinclair et al., 2002; Sarfraz et al., 2020).

Gordon (2006) explains that the rational cognitive structure utilizes the cognitive parts of entrepreneurs to contemplate and even clarify their behavior, which is associated with their chances of organization, creation, and business development portrayed by TPB. Indeed, the term “cognitive style” is utilized to describe certain methods of handling data, identified as being aligned with entrepreneurial behavior. Few researchers recognize the information structures that entrepreneurs use to make appraisals, decisions or choices, assess new openings and create and develop organizations (Sánchez, 2009).

Different sorts of examinations depend on what an individual thinks, says, or does; these affect the cognitive cycles through which people obtain, use, and interact with data (Krueger, 2017). Zhao et al. (2012) contend that entrepreneurs think and interact with data uniquely in contrast to non-entrepreneurs. Such contrasts might assist with recognizing individuals who establish or plan to build up organizations (entrepreneurs) from individuals who do not (non-entrepreneurs). In this manner, a few creators have used the term “cognitive style” to portray certain methods of handling data identified with entrepreneurial behavior (Bouckenooghe et al., 2005).

Allison et al. (2000) argue that senior managers have cognitive styles similar to those of entrepreneurs. The studies of Lindblom et al. (2008) have discovered different cognitive styles of entrepreneurs. The TPB structure is generally acknowledged as the best way of considering entrepreneurial intention, which develops behavior (Schlaegel and Koenig, 2014). The theory focuses on cognitive styles of an individual, which indicates the cognitive capability of an individual who wants to become an entrepreneur. Therefore, this study proposes that:

Hypothesis 1 (a): Rational cognitive styles have a positive influence on entrepreneurial behavior.
Hypothesis 1 (b): Experiential cognitive styles have a positive influence on entrepreneurial behavior.

### Cognitive Style and Entrepreneurial Intention

“Act tendency,” “perceived possibility,” and “perceived interest” are the powerful factors identified by Krueger et al. (2000) as affecting entrepreneurial intention. Bird (1988) indicates that rational cognitive style can be measured from those elements that most impact the intention. Several researchers have investigated the intention model and applied new factors that impact the intention, e.g., cognitive style. Bird explains that thoughts of an individual start with motivation. However, intention and attention are needed to understand the thoughts of the individual; we must accept that intention is a mixture of goals and objectives, insights, cause–impact thinking, and natural, comprehensive, and logical reasoning. For example, the Shapiro model measures demographics like gender and country to understand the undergraduate intentions of those with experiential cognitive styles (Krueger, 2017). In light of the TPB, it is clear that individuals have different cognitive styles. While an individual should select one cognitive style by choice, in general, each style has a different impact on human judgments. It all depends on the difficulty of the work, environment, and individual contrasts (Schlaegel and Koenig, 2014). This implies that people can shift among the different cognitive styles. Accordingly, it is feasible to prepare people to adopt a specific method of cognitive style contingent upon the unique circumstance or depending on the difficulty of the work (Sarfraz et al., 2018). Ornstein (1972) describes that those cognitive styles are separate elements of mindfulness that predict different sides of people. Hodgkinson and Sadler-Smith (2003) contend that all individuals with different cognitive styles need to be treated differently on separate, unipolar scales.

The primary attributes of the rational style are: formal and arranged, appearing through extreme cognitive exertion, and being aware of everything. Formal arranging and asset control are required when there is some need for it, there are variations in the situation, and the amount of data is increasing (Brinckmann et al., 2010). Think about arranging different processes; generally, this arranging needs to be continually updated. In this manner, changes can affect the primary goals (Randerson et al., 2016).

Experiential styles are characterized by a person having a preaware, complete, and quick action plan. Mitchell et al. (2007) explain that experiential style is a unique one in which sharp entrepreneurial cognitions connect with spatial skills (e.g., industry, explicit conditions, innovation, and culture) to create awareness of a chance to make new worth. So, the primary attributes of experiential styles are as follows: begins with the past cognizant idea, incorporates complete affiliations, and outcomes in emotionally charged decisions (Blume and Covin, 2011). Therefore, this study proposes:

Hypothesis 2 (a): Rational cognitive styles have a positive influence on entrepreneurial intention.
Hypothesis 2 (b): Experiential cognitive styles have a positive influence on entrepreneurial intention.

### Entrepreneurial Intention and Entrepreneurial Behavior

A set of direct and indirect activities are identified with the start-up of additional projects and the search for and assessment of new opportunities called entrepreneurial behavior. The intention is a basic model for entrepreneurial behavior development and is the best indicator of human behavior. Drucker (2014) contends that entrepreneurship is behavior and, for the most, is practiced willfully; however, as Prophet Mohammad (P.B.U.H) said, “People's actions depend on their intentions.” In this manner, before any entrepreneurial activity or behavior occurs, its aim is framed. Intentions predict entrepreneurial behavior in an ideal manner.

In the same way, a rise in intention possibly relates to effective entrepreneurial action. Accordingly, entrepreneurship training primarily targets the creation and support of entrepreneurial expectations among energetic entrepreneurs, mainly undergraduate students. Thus, many studies conducted in different countries (e.g., Iran, USA, Malaysia, Russia, and India) measure intentions of undergraduate students (Zali et al., 2012). Kume et al. (2013) explain that it is a serious requirement for universities to create entrepreneurial attitudes among their undergraduates through targeted activities and to attempt to incorporate skilled knowledge and professional abilities to educate entrepreneurial graduates. The TPB structure shows that entrepreneurial thoughts start with motivation. However, intention and attention are needed to understand entrepreneurial thoughts. As such, the intention is a mixture of goals and objectives, insights, cause–impact thinking, and natural, comprehensive, and logical reasoning. Similarly, the intention is a vital factor that assumes a significant role in moving from intention to entrepreneurial behavior. Therefore, this study proposes that:

Hypothesis 3: Entrepreneurial intention has a positive influence on entrepreneurial behavior.

### Mediating Effect of Entrepreneurial Intention

The rational cognitive style addresses contrast in intellectual behavior of an individual (Kickul et al., 2009). People with a rational cognitive style can evaluate various social standards after seeing the achievement and attractiveness of opening a new trade. They are fairly practical, although for them opportunity has some value and is a significant motivational factor. They need adequate energy and enthusiasm to change from observed opportunity level to intention. Therefore, intention mediates between cognitive styles and entrepreneurial behavior. People with experiential cognitive styles are high-risk-takers and more excited to grasp opportunities (Barbosa et al., 2007).

Cognitive structures are not just a guide to understanding people and their behavior and considering their psychological cycles when they connect with others, they also address the climate in which these psychological cycles and associations occur (Mitchell et al., 2002). Therefore, specialists demand the ability to clarify a significant part of entrepreneurial behavior and its starting point from both cognitive styles (Sánchez et al., 2011). Rational cognitive styles address and contain information, while experiential cognitive styles identify how we collect information and utilize it. Therefore, the field of entrepreneurial cognition incorporates all parts of cognition that might play a major role in specific parts of the entrepreneurial interaction (Baron and Markman, 1999).

Similarly, high-risk takers did not display greater levels of affiliation, administrative self-efficacy, and resilience self-efficacy. Therefore, people with experiential cognitive style were sure about their capacity to distinguish between and perceive opportunities, albeit without a lot of trust in their appraisal, assessment, arranging, and marshaling of assets abilities (Kickul et al., 2009). However, people with the rational cognitive style were more optimistic about their capacities to assess, survey, marshal assets, and plan. Still, they felt less positive about their capacities to look for and perceive new openings. Kickul et al. (2009) found that a person who wants to become a successful entrepreneur has the power to be decisive. The experiential cognitive styles focus on the impact of the said impressions and understanding the situation. In entrepreneurial cycles, Baron (2006) recommends that to distinguish a chance, entrepreneurs utilize a cognitive structure to process recently procured information and relate it to the concept in terms of their own discrimination and translation of changes in the outside climate through their thinking and experience. Mitchell et al. (2007) state that individuals who want to become entrepreneurs need to maximize their cognitive style. First, those entrepreneurs who work in conditions with a high degree of vagueness and difficulty, and have an overloaded and rational cognitive style, are required to make decisions and become successful entrepreneurs (Jin et al., 2017). Second, Frese and Gielnik (2014) address experiential cognitive styles that may expand intention because of cognitive biases, e.g., data determination representativeness, the law of slight numbers, inappropriateness, and overoptimism. Hence, it is predictable that undergraduate students using different cognitive styles will build a positive intention, which will lead to entrepreneurial behavior. As such, this study proposes:

Hypothesis 4 (a): Entrepreneurial Intention mediates the relationship between rational cognitive styles and entrepreneurial behavior.
Hypothesis 4 (b): Entrepreneurial Intention mediates the relationship between experiential cognitive styles and entrepreneurial behavior.

### Moderating Effect of Informal Learning

In the advanced workplace, firms and employees are presented with and rapidly build their own novel thoughts, innovations, administrations, and guidelines. Proactive and intelligent firms and staff should be interested not only in individuals who bring abilities and information (from formal training) to the work environment, but also those who can learn, share, and create information at work, inside the work environment. Researchers have perceived the significance of informal learning in the workplace for a long time at the individual level (Eraut, 2010). According to Kickul et al. (2009), entrepreneurial research reveals that the use of rational cognitive styles is more suitable for handling data completely, including drawing an obvious conclusion regarding data by using informal learning concerning assets that assist people in starting or developing a new business (e.g., connections, resources, and contacts), and perceiving opportunities based on that informal learning that many people may disregard. Therefore, experiential cognitive styles are bound to upgrade awareness to assist with ensuring the required assets and taking advantage of new market openings.

Moreover, the rational cognitive style is useful for guiding individuals with more cognitive differences to miscalculate their capacities to make an effective entrepreneur (Cassar, 2010). For instance, experiential cognitive styles can bring about carelessness, fantasies of control (Frese and Gielnik, 2014), and complex unthinkingness (Wiklund et al., 2018), which improve only when people have faith in their capacity to prevail and become successful entrepreneurs. Therefore, this study proposes that:

Hypothesis 5 (a): Informal learning moderates the relationship between rational cognitive styles and entrepreneurial intention.
Hypothesis 5 (b): Informal learning moderates the relationship between experiential cognitive styles and entrepreneurial intention.

**Figure 1:**
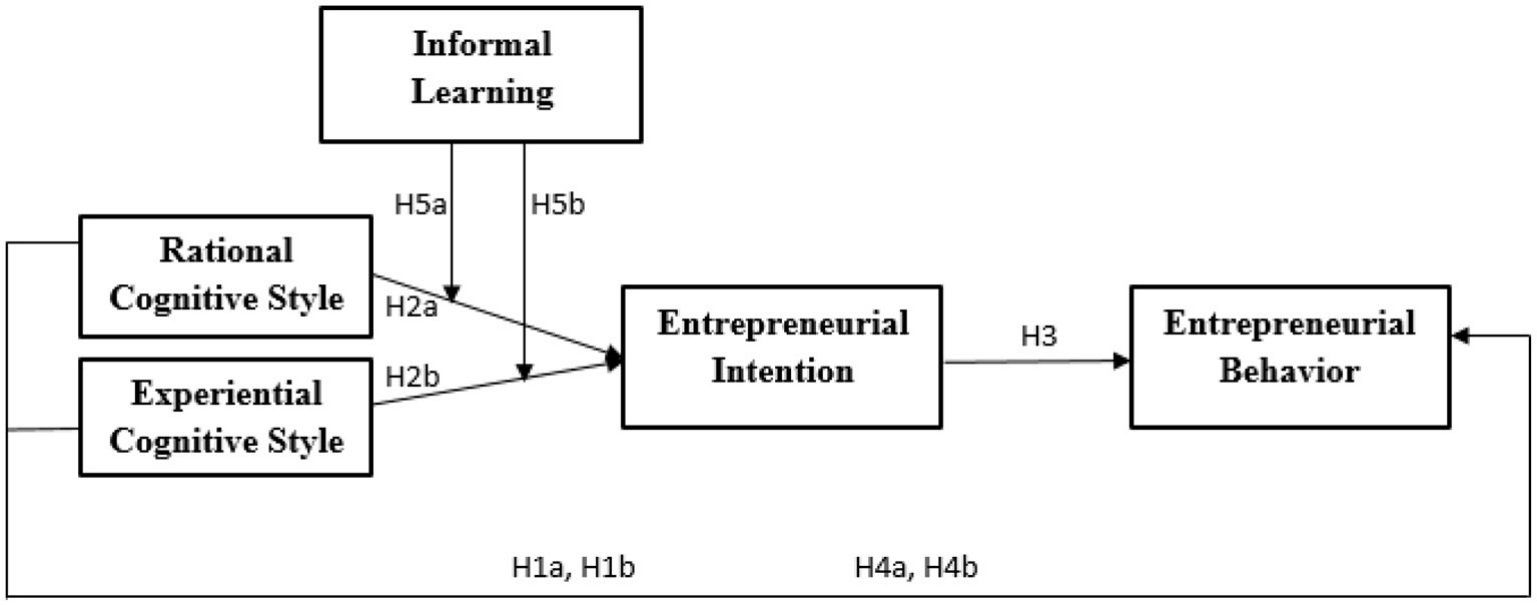
Research model.

Figure 1 shows study hypothesis and variables relationship.

## Research Methodology

Considering the scarcity of research in emerging and developing economies like Pakistan and the lack of empirical studies exploring entrepreneurial dynamics of Pakistan, this article aims to perform a wider field investigation of entrepreneurial cognition and entrepreneurial behavior. Using convenience sampling techniques, a self-administered survey was conducted. Data was collected in February 2021 and March 2021 from final year project students at universities located in Islamabad and Rawalpindi, Pakistan. The twin cities, Islamabad and Rawalpindi, are in metropolitan areas that are home to around 4.1 million inhabitants (Ishtiaq et al., 2017). Rawalpindi is an ancient city with high density, whereas Islamabad, planned and developed in the 1960s, is an administrative and residential city with medium density. The Metropolitan Area (RIMA) of Rawalpindi–Islamabad is typically considered an urban area, and about 70,000 employees and students travel between the two cities every day (Adeel et al., 2016). Students from almost every city of Pakistan attend universities located in the twin cities.

Hence, based on their characteristics of being metropolitan cities and the limitation of the author to travel to different cities for data collection during the COVID-19 pandemic, we decided to collect responses from students of four public universities located in the twin cities (i.e., two from Islamabad and two from Rawalpindi) that have a functioning business incubation center (BIC). These centers were established based on the Higher Education Commission BIC program for public universities, whose sole purpose is to promote entrepreneurial activities within the institute and develop entrepreneurship behavior among students.

According to Podsakoff et al. (2003), a time-lagged study is suggested to control common method biases. Therefore, for the current research, data was collected in three different periods, with a gap of 15 days between each time. Responses on cognitive styles (rational and experiential) and informal learning were collected at time 1; entrepreneurial intention at 2; and entrepreneurial behavior at 3. Initially, at time 1, 400 students were contacted and asked about demographic, independent, and moderating variables, i.e., rational cognitive styles and experiential cognitive styles and informal learning. Of these, 372 usable questionnaires were received, i.e., a response rate of 93%. After a gap of 15 days, i.e., at period 2, the 372 students who gave complete responses were contacted again and asked about their entrepreneurial intention. This resulted in a 90% response rate, i.e., 338 usable responses were received. For time 3, only those participants who participated at periods 1 and 2 were revisited and requested to complete the survey containing items on entrepreneurial behavior. A total of 320 complete responses were received, with an overall response rate of 80%.

For research, the minimum sample size is recommended to be five times the number of items (Bryant and Yarnold, 1995) and (Norusis, 2005) suggested that the study included a minimum of 300–350 responses to conduct analysis. Keeping in view the above-mentioned past suggestion of the methodologist, the current study analyzed 320 complete responses, which was finalized after data screening and cleaning. Hence, 400 questionnaires were distributed, of which 320 complete responses were received with a response rate of 80%. The convenience sampling technique was adopted for this research because it is affordable and respondents are freely and easily available. Moreover, it fulfills the basic assumption related to the convenience sampling technique, the target population is homogenous. As such, no effect will be incurred on the study results if the data is collected from a “random sample, a nearby sample, a co-operative sample, or a sample gathered in some inaccessible part of the population” (Etikan et al., 2016).

Demographic statistics revealed 42% of respondents were female, and 58% were male. Of the respondents, 68% were undergraduate students and 32% were at the graduate level. Regarding their education, 48% of respondents were enrolled in a management sciences degree, 27% were from the engineering department, and 25% were from the computer sciences department (see Table 1).

**Table 1:** Demographics statistics.

**S No**.	**Demographics**	**Categories**	**Percentage**
1	University Level	Undergraduate Level	68%
		Graduate Level	32%
2	Gender	Male	58%
		Female	42%
3	Department	Management Sciences	48%
		Engineering	27%
		Computer Sciences	25%

### Measures

Relational and experiential cognitive styles were measured by using a 24-item scale developed by (MacCallum and Austin, 2000). Sample items include: “I want to have a full understanding of all problems;” “I like to analyze problems;” “I prefer well-prepared meetings with a clear agenda and strict time management;” “I like to contribute to innovative solutions;” “New ideas attract me more than existing solutions.” The scale was measured using a five-point Likert scale from strongly disagree to agree strongly. To measure informal learning, a 10-item scale suggested by Pereira et al. (2019) was used. Respondents were asked about “To what extend you observe family and friends' action and try to replicate;” “To what extend you seek for help (friends and family) when in doubt or trouble.” The scale was measured using a five-point Likert scale from strongly disagree to agree strongly. The entrepreneurial intention was measured using a 6-item scale by Liñán and Chen (2009). Sample items include: “I am ready to do anything to be an entrepreneur;” “I have very seriously thought of starting a firm.” Entrepreneurial behavior was measured using an 18 item scale developed by (Rauch and Hulsink, 2015). Sample items include: “Spent a lot of time thinking about starting a business?;” “Organized a start-up team?” and “Defined market opportunities?”

### Confirmatory Factor Analysis

After data screening and cleaning, to check the validity of the scale, confirmatory factor analysis (CFA) was conducted. Five-factor model CFI = 0.965; GFI = 0.904; AGFI = 0.893; NFI = 0.918; RMSEA = 0.04 was compared with one-factor model CFI = 0.814; GFI = 0.799; AGFI = 0.682; NFI = 0.758; RMSEA = 1.32. Results of the CFA revealed that fit indices of the five-factor model are better and within the acceptable range as compared with the one-factor model, thereby confirming discriminant validity of the variables.

## Study Results

Table 2 illustrates descriptive and correlation analysis, average variance extracted, Cronbach alpha, and composite reliability. As shown, relational cognitive style is significantly related to experiential cognitive style (*r* = 0.495, *p* < 0.05), entrepreneurial intention (*r* = 0.491, *p* < 0.05), informal learning (*r* = 0.587, *p* < 0.05), and entrepreneurial behavior (*r* = 0.488, *p* < 0.05). To check validity of the study variable, average variance extracted (AVE) was calculated; moreover, internal consistency reliability (Cronbach alpha) and composite reliability was also conducted; and the result depicts values within range as suggested by Hair (2010).

**Table 2:** Mean, SD, correlation, reliability, and validity.

		**Mean**	**ICR**	**CR**	**AVE**	**1**	**2**	**3**	**4**	**5**
1	Relational cognitive style	4.08	0.702	0.710	0.551	1				
2	Experiential cognitive style	4.07	0.776	0.789	0.598	0.459^**^	1			
3	Informal learning	3.85	0.842	0.852	0.532	0.587^**^	0.548^**^	1		
4	Entrepreneurial intention	3.94	0.791	0.798	0.685	0.491^**^	0.650^**^	0.463^**^	1	
5	Entrepreneurial behavior	3.75	0.771	0.782	0.657	0.488^**^	0.671^**^	0.483^**^	0.662^**^	1

*n = 320; ICR; Internal Consistency reliability (Cronbach's alpha); CR, Composite Reliability; AVE, average variance extracted*.

** *p < 0.01*.

### Hypothesis Testing

Table 3 shows the results of H1, H2, H3, and H4 (i.e., mediation analysis). Hypothesis 1 states that relational, cognitive style (H1a) and experiential cognitive style (H1b) is positively related to entrepreneurial behavior and is shown to be significant with β = 0.522, *p* < 0.001 and β = 0.685, *p* < 0.001. Hypothesis 2 states that relational, cognitive style (H2a), and experiential cognitive style (H2b) is positively related to entrepreneurial intention and is shown to be significant with β = 0.430, *p* < 0.001 and β = 0.385, *p* < 0.001 respectively. Hypothesis 3 states that entrepreneurial intention is positively related to entrepreneurial behavior and is shown to be significant with β = 0.346, *p* < 0.001; hence hypotheses 1,2, and 3 are statistically proved. For mediation analysis, indirect effects were calculated; results in Table 2 indicate that entrepreneurial intention mediates the relationship between relational and experiential cognitive style and entrepreneurial behavior; as “bootstrapped confidence interval does not include Zero” (as shown in Table 3). Hence hypotheses 4a and 4b are approved.

**Table 3:** Mediated regression analysis results.

**Relationships**	**Effect**	**SE**	***T***	***p***
RCS → EB	0.522	0.125	11.122	0.000
ECS → EB	0.685	0.235	9.565	0.000
RCS → EI	0.430	0.309	15.550	0.000
ECS → EI	0.385	0.328	13.864	0.000
EI → EB	0.346	0.313	10.530	0.000
	**Effect**	**SE**	**LL 95% CI**	**UL 95% CI**
**Bootstrap results for indirect effects**				
Indirect Effects (RCS, EI, EB)	0.148	0.287	0.1164	0.2281
Indirect Effects (ECS, EI, EB)	0.133	0.256	0.0155	0.0345

*n = 320; RCS, Relational cognitive style; ECS, Experiential cognitive style; EI, Entrepreneurial intention; EB, Entrepreneurial Behavior. Bootstrap Sample Size, 5,000. LL, Lower Limit; CI, Confidence Interval; UL, Upper Limit*.

**Figure 2:**
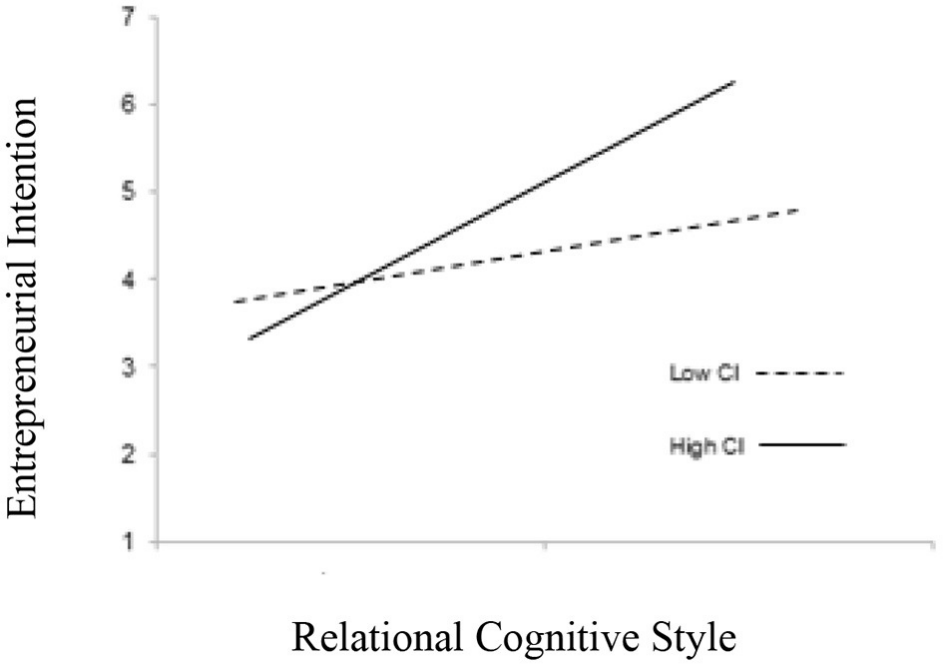
Interaction effects of relational cognitive style and informal learning on entrepreneurial intention.

**Figure 3:**
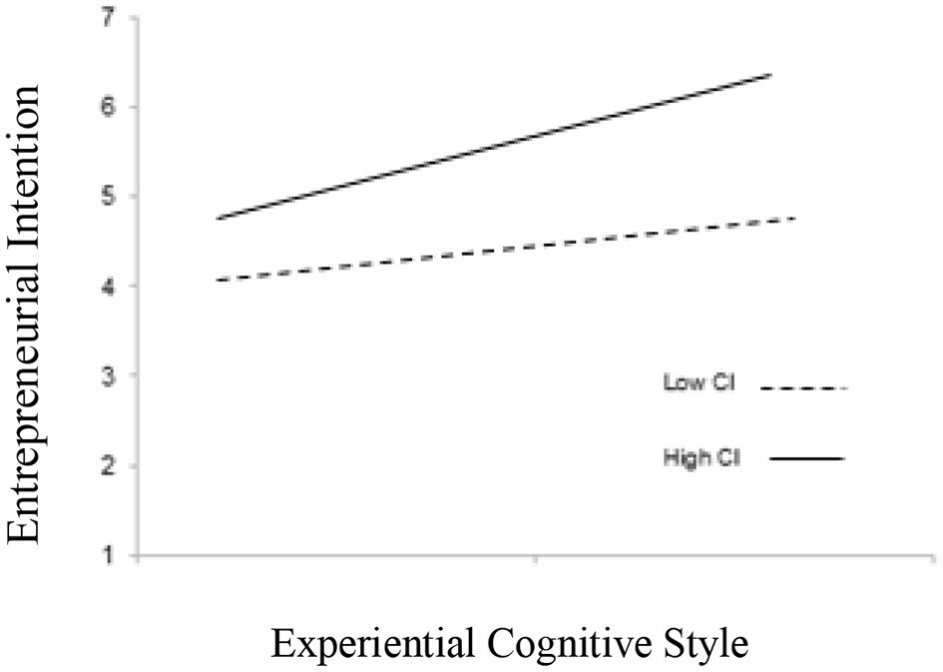
Interaction effects of experiential cognitive style and informal learning on entrepreneurial intention.

In line with H5, the interaction term of relational (H5a), experiential cognitive style (H5b), and informal learning for the entrepreneurial intention were found significant (β = 0.121. *p* < 0.05) and (β = 0.091. *p* < 0.01). Moreover, Table 4 illustrates that the relationship between relational, cognitive styles, and entrepreneurial intention strengthens in the case of high informal learning (β = 0.482, *p* < 0.001), as compared with low informal learning (β = 0.102, *p* < 0.001). Similarly, experiential cognitive styles and entrepreneurial intention strengthens in the case of high informal learning (β = 0.530, *p* < 0.001), as compared with low informal learning (β = 0.256, *p* < 0.05) (see Figures 2, 3). Hence, hypotheses 5a and 5b are accepted.

**Table 4:** Hierarchical moderated regression analysis.

**Predictors**	**Entrepreneurial intention**			
	**R**	**R^**2**^**	**Estimate**	**SE**
Step1	0.32^***^	0.21^***^		
Constant			5.10	0.08
RCS			0.45^**^	0.12
ECS			0.37^**^	0.10
IL			0.27^**^	0.09
Step 2	ΔR^2^	0.03		
RCS x IL			0.121^*^	0.16
ECS x IL			0.091^***^	0.08
**Moderator**	**Entrepreneurial Intention**			
**IL**	**Effect**	**Boot SE**	**LLCI**	**ULCI**
**Conditional direct effects of X (RCS) on Y (EI) at values of the moderator (i.e., IL)**				
IL−1SD	0.102^***^	0.08	0.15	0.31
IL mean	0.330^**^	0.10	0.04	0.50
IL +1SD	0.482^***^	0.15	0.19	0.30
**Conditional direct effects of X (ECS) on Y (EI) at values of the moderator (i.e., IL)**				
IL−1SD	0.256^*^	0.13	0.17	0.21
IL mean	0.410^**^	0.19	0.06	0.10
IL +1SD	0.530^***^	0.21	0.22	0.35

*n = 320; RCS, Relational cognitive style; ECS, Experiential cognitive style, IL, Informal learning; EI, Entrepreneurial intention; EB, Entrepreneurial Behavior. Bootstrap Sample Size, 5000. LL, Lower Limit; CI, Confidence Interval; UL, Upper Limit*.

* *p < 0.05*,

** *p < 0.01*,

*** *p < 0.001*.

## Discussion

Entrepreneurial cognition is an essential element of the entrepreneurial process that explains how entrepreneurs can be modeled (Mitchell et al., 2007). According to Armstrong et al. (2012) and Chen et al. (2015), individual behavior is determined by cognitive style; it explains how individuals think, behave, make decisions, and solve problems. Therefore, investigating the role of entrepreneurial cognition in determining successful entrepreneurship is a significant concern (Allinson et al., 2000). Besides its importance, little research has been conducted to examine how cognitive styles of individuals determine entrepreneur behavior (Chen et al., 2015).

By utilizing the theory of planned behavior and reviewing the literature on the psychological perspective in entrepreneurship, the current study sheds light on the existing literature. It proposed a research framework that explains how and why cognitive style influences entrepreneurial behavior. Our findings illustrate that cognitive style develops entrepreneurial intention and behavior among students, thereby proving hypotheses 1, 2, 3, and 4. Further, informal learning through connections and surroundings may assist students in identifying an opportunity, developing entrepreneurial intention, and starting a new business, thereby proving hypothesis 5.

### Theoretical Implications

This study contributes to the knowledge of the field of the positive role of the rational and experiential cognitive styles in the entrepreneurship dynamic. First, our research on cognitive style identified the vital finding that both rational and experiential cognitive styles have an immediate beneficial outcome on entrepreneurial behavior. This contrasts with previous research, which categorizes people as showing either rational cognitive style or not, or only evaluates which one of the two cognitive styles expands entrepreneurial behavior (Kickul et al., 2009). Conversely, our study shows that both cognitive styles might positively affect entrepreneurial behavior.

Second, our study expands the use of the TPB to entrepreneurial behavior research and finds that cognitive style undoubtedly impacts entrepreneurial behavior and intention. TPB allowed us to gain a more extensive comprehension of the components that influence entrepreneurial behavior (Schlaegel and Koenig, 2014). Our research is one of few that adopts this approach. Further research may analyze other individual-level qualities that upgrade or restrain entrepreneurship instruction on new and novel approaches to improve entrepreneurial ambitions of undergraduates.

Third, our study showed that cognitive styles assumed a more significant place in forming behavior of undergraduates than expected. Our findings suggest that cognitive style assumes a more significant part in deciding the behavior in specific societies. At the same time, in different nations, the impact of context-oriented factors like guidelines, social qualities, and institutional variables might strongly affect the choice to be an entrepreneur (Schlaegel and Koenig, 2014). In this way, we suggest that the significance of individual-level factors, such as cognitive style on the perspectives of undergraduates and discrimination toward entrepreneurship, also contrasts across nations. Further research is expected to foster an extensive perspective on the role of organizations and public approaches on the mentalities of the undergraduates and their perceived behavioral control over a new business (Schlaegel and Koenig, 2014). Such studies will provide important insights that will assist institutions to enhance the behavior of undergraduates.

### Practical Implications

According to a practical perspective, investigating the cognition and factors identified with entrepreneurial behavior and intention in the twin cities (Islamabad and Rawalpindi) reveals how authorities can implement effective techniques to inspire undergraduates and graduates to become entrepreneurs. As an initial step, our study proposes that instructors might try to comprehend the cognitive styles of undergraduates. Next, targeted interventions might be feasible, including creating or improving rational and experiential cognitive styles by preparing and communicating data to undergraduates in manners that match their cognitive style and grouping undergraduates with various cognitive styles to improve their learning accordingly. Moreover, undergraduates might look for the help of others who have the fundamental cognitive styles required for entrepreneurship action.

This research was directed practically, i.e., to develop further entrepreneurship training in the twin cities (Islamabad and Rawalpindi). Recently, individuals (younger than 30) have consistently requested more prominent freedom and less government impedance in the twin cities. With the ongoing changes occurring in cities in Pakistan in mind, entrepreneurial action around the world can also be strengthened by inventing adopting new and inventive monetary advancement techniques and approaches. Witnessing how young people can see new opportunities and settle on professional decisions will improve the capacity of governments to plan viable financial and instructive projects. These projects can direct and guide youngsters more compellingly, and consequently lessen the relentless issue of joblessness influencing the twin cities.

Informed by our study, we also recommend that the mediations used to foster goals might vary by culture. For instance, our outcomes propose that to motivate undergraduates to launch a business, entrepreneurship instruction in the twin cities might need to focus fundamentally on changing the cognitive styles of undergraduates and entrepreneurial/business abilities. For instance, entrepreneurship training in twin cities might have to dedicate more focus toward addressing and maybe provoking the longing of undergraduates to adjust to the cultural benefits of seeking wellbeing and avoiding vulnerability. Meanwhile, in nations with higher context-oriented obstacles, placing a more prominent spotlight on exploring institutional and administrative obstructions may be more applicable in empowering undergraduates to begin a business.

This research validates and considers the current heterogeneity in entrepreneurship undergraduates regarding their cognitive styles and social impacts. Our findings offer a brief look at what parts of entrepreneurship instruction might not have been successful while providing advice regarding how instructors could improve their methodology. Overall, our discoveries highlight that there is no size-fits-all answer for encouraging the entrepreneurial aims of the undergraduates.

### Study Limitations/Future Research Directions

This research finds that cognitive styles play a significant role in research regarding entrepreneurs. Entrepreneurs have been ascribed a different limit to other professionals regarding the ability to prepare data, and the idea of entrepreneurial cognition has been touted as an unmistakable element that characterizes entrepreneurs. Nevertheless, this analysis is quite simplified; that is, it does not consider factors external to the person when trying to clarify entrepreneurial behavior, or even within the entrepreneur themselves, though some prior research does present context-oriented factors. Research on the cognitive role in entrepreneurial interactions has commonly centered around several cognitive factors and explicit periods of the entrepreneurial process. This is why we have no intelligible, coordinated models that offer an indisputable view on the significance of cognitive foundation of a person and its advancement throughout the entrepreneurial process. Our literature review also reveals that many studies, since they are centered around considering the cognitive capacities of business makers, focus on the idea of an entrepreneur as a “whole,” leaving to the side different types of entrepreneurs. The cognitive direction is a somewhat new field in the investigation of entrepreneurship. Consequently, it is conceivable that it has a lot to contribute, while it is likewise evident that analysts need to expand their focus.

The first limitation of the study is that we did not inspect the outcome variable of launching a business. However, earlier examinations have shown that entrepreneurial behavior is associated with opening a new business later on, so future research may investigate execution results, e.g., of a startup. Second, we did not quantify different cultures. As surveys likely reveal contrasts in culture among people and the singular impacts of each social measurement, this data would have been useful. Further, it may be significant to consolidate tests from various nations to evaluate how the connections might vary across nations.

Finally, our research is dedicated to assessing the effects of cognitive styles on the expectation of a person that they will begin a business later on. Future investigations might study the cognitive role during a few distinct stages in the entrepreneurial cycle. For instance, it might be the case that experiential cognitive style is beneficial during the chance revelation, idea generation, and recognizable proof stages, whereas rational style might flourish during the arranging, attainability examination, and idea-execution stages.

## Conclusion

The cognitive styles of individuals are the main elements to predict the establishment of start-up businesses in the future by undergraduate students. However, aside from styles, entrepreneurial thoughts of students and learning from their surroundings informally support their intentions. Hence, one of the objectives for entrepreneurial education is for projects to be outlined and conducted in a manner that builds intentions of the students. On the other hand, revelatory thoughts inspire undergraduates to develop new organizations because of their interesting nature. As per the results, rational cognitive styles have a direct impact on the intention of undergraduate students. Similarly, the experiential cognitive style has an effect on framing the entrepreneurial behavior of undergraduate students, and rational cognitive style impacts behavior through intention. Hence, this study is significant because it explains that the relationship between cognitive styles (rational and experiential) and entrepreneurial behavior is mediated through the intentions of the undergraduate students, whereas informal learning moderates the relationship between cognitive styles (rational and experiential) and intention.

## Data Availability Statement

The raw data supporting the conclusions of this article will be made available by the authors, without undue reservation.

## Ethics Statement

Ethical review and approval was not required for the study on human participants in accordance with the local legislation and institutional requirements. The patients/participants provided their written informed consent to participate in this study.

## Author Contributions

All authors listed have made a substantial, direct and intellectual contribution to the work, and approved it for publication.

## Funding

This study was supported by Hundreds of Schools Unite with Hundreds of Counties-University Serving Rural Revitalization Science and Technology Support Action Plan (Grant no. BXLBX0847), Hubei Self-Science Fund Project (Grant name: brain tumor diagnosis based on capsule neural network), and National Statistical Science Research Project in 2020, China (Grant no. 2020LY023).

## Conflict of Interest

The authors declare that the research was conducted in the absence of any commercial or financial relationships that could be construed as a potential conflict of interest.

## Publisher's Note

All claims expressed in this article are solely those of the authors and do not necessarily represent those of their affiliated organizations, or those of the publisher, the editors and the reviewers. Any product that may be evaluated in this article, or claim that may be made by its manufacturer, is not guaranteed or endorsed by the publisher.
